# Effectiveness of an Attachment-Based Intervention Program in Promoting Emotion Regulation and Attachment in Adolescent Mothers and their Infants: A Pilot Study

**DOI:** 10.3389/fpsyg.2016.00195

**Published:** 2016-02-24

**Authors:** Cristina Riva Crugnola, Elena Ierardi, Alessandro Albizzati, George Downing

**Affiliations:** ^1^Department of Psychology, University of Milano-BicoccaMilan, Italy; ^2^ASST Santi Paolo and Carlo HospitalMilan, Italy; ^3^Pitié-Salpêtrière HospitalParis, France

**Keywords:** adolescent mother, mother-infant interaction, dyadic affective coordination, maternal attachment, video intervention

## Abstract

This pilot study examined the effectiveness of an attachment-based intervention program, PRERAYMI, based on video technique, psychological counseling and developmental guidance in improving the style of interaction and emotion regulation of adolescent mothers and their infants after 3 and 6 months of intervention. Analyses revealed that adolescent mothers who participated in the intervention (vs. control group adolescent mothers) increased their Sensitivity and reduced their Controlling style after both 3 and 6 months of treatment. Infants who participated in the intervention (vs. control group infants) increased their Cooperative style and reduced their Passive style from 3 to 9 months. Moreover, the intervention group dyads (vs. control group dyads) increased the amount of time spent in affective positive coordination states (matches), decreased the amount of time spent in affective mismatches, and had a greater ability to repair mismatches from 3 to 9 months. Furthermore, the intervention group dyads (vs. control group dyads) increased the amount of time spent in reciprocal involvement in play with objects from 3 to 9 months. The quality of maternal attachment did not affect the intervention effect.

## Introduction

Early motherhood is considered a significant risk factor for the establishment of an adequate relationship between mother and infant (Osofsky et al., 1993; Pomerleau et al., 2003) and for the subsequent developmental trajectories of both mothers and infants. Adolescent mothers' management of their parental role is, in fact, interfered with by problems relating to their transition to adulthood, involving processes of individuation from parent figures (Fraiberg, 1978; Aiello and Lancaster, 2007). This developmental task can easily conflict with their taking on a parental role. The newly born's strong need for physical and emotional care competes with the adolescent mother's needs (Reid and Meadows-Oliver, 2007). This may create strong conflict in the young mother between her need for autonomy and the infant's intense dependency on her, giving rise to depression, parenting stress and low self-esteem and affecting her ways of relating to the infant (Osofsky et al., 1993; Reid and Meadows-Oliver, 2007; Secco et al., 2007). Moreover, an adolescent mother's cognitive and neurophysiological development still has to be completed (Giedd, 2005). Mothers under 20 are less cognitively competent with regard to taking on their parental role (cognitive readiness to parent) and to knowledge of the stages of development of their infants (Whitman et al., 2001).

Furthermore, motherhood in adolescence is often associated with other risk factors correlated to poor parenting, such as low socio-economic status (SES) and educational attainment. However, a number of studies have shown that, even when the effect of such variables is controlled, adolescence is still, *per se*, a high risk factor for a mother's parenting skills (Bornstein et al., 2006; Rafferty et al., 2011). Other risk factors in the post-partum period may also be associated with being an adolescent mother, such as post-natal depression—50% more adolescent mothers are depressed than adult mothers—(Brown et al., 2012) and being a single mother (Logsdon et al., 2005). A further important risk factor for mother-infant interaction (Riva Crugnola et al., 2013a) and for the development of secure attachment in the infant (Main, 1995) is that adolescent mothers are more likely to have insecure attachment models than adult mothers (Madigan et al., 2006; Riva Crugnola et al., 2014) and to have experienced physical and sexual abuse in their lives (Madigan et al., 2012).

The concurrence of various risk factors for parenthood together with the conflict between different developmental tasks makes the adolescent mother-infant relationship difficult right from the very beginning. In caring for their infants adolescent mothers use more instrumental behavior (Krpan et al., 2005) and are more intrusive toward their children, displaying poor emotional availability compared to adult mothers (Easterbrooks et al., 2005). Dyadic emotional regulation is also less adequate than it is between adult mothers and infants, related to the difficulty of the mothers in regulating the negative emotions of their infants (Riva Crugnola et al., 2014). Compared to adult mothers, adolescent mothers show both poorer ability to scaffold the activity of their infants (Easterbrooks et al., 2005) and are lower in mind-mindedness (Demers et al., 2010).

These characteristics of the relationship between adolescent mothers and their infants, together with the above risk factors, linked to early parenthood leads to problematic outcomes for both in the short and long term. In the short term the infants may display delays both in their psychomotor development (Jahromi et al., 2012) and in their cognitive development (Bolton, 1990; Morinis et al., 2013). They also show a greater tendency to construct insecure avoidant and disorganized attachment ties than do the children of adult mothers (Broussard, 1995; Lounds et al., 2005) and have a greater probability of suffering abuse by their young mothers. Furthermore, in adolescence and adulthood they display a range of adverse outcomes, such as poor academic achievement, behavior problems, early parenthood, and violent offending (Jaffee et al., 2001; Hoffman and Maynard, 2008). Early motherhood, at the same time, limits the subsequent life opportunities of young women (Jaffee et al., 2001), leading them to attain only low levels of education and to have a higher probability of suffering depression and social isolation (Horwitz et al., 1996; Boden et al., 2008).

### The intervention program

#### Aims and methods

The difficulties inherent in initial relations between young mothers and their infants and the impact of this experience on the developmental trajectories of both led us to design an attachment-based intervention program for adolescent mothers and their infants, entitled “Promoting responsiveness, emotion regulation and attachment in young mothers and infants” (PRERAYMI; Riva Crugnola et al., 2013b).

There have been various programs aimed at making the relationship between adolescent mothers and infants more adequate (Savio Beers and Hollo, 2009). Some of these programs are specifically attachment-based, i.e., aimed at increasing the responsiveness of mothers and thus improving the quality of infant attachment. These include the pioneering program of Carter et al. (1991) “Speaking for the Baby,” which uses video-feedback and aims to give a voice to infant communication, which is often either not understood or misunderstood by young mothers. Of particular interest is also the MTB (Minding the Baby) mentalization-based intervention which combines the home visiting approach with the use of video-feedback. Its main aims are to increase the sensitivity and reflective function of young mothers and to promote secure attachment in the infant (Slade et al., 2005b; Sadler et al., 2013). Moran et al. (2005) also devised a brief intervention program to promote adolescent mothers' sensitivity which is based on an integration of home visiting and video-feedback technique.

Our program draws inspiration from these attachment-based programs. The PRERAYMI is an attachment based medium-term intervention program for adolescent and young mothers (aged between 14 and 21), their partners and their infants. It is an academic community partnership program, the result of collaboration between the Infant Neuropsychiatric Unit of the San Paolo Hospital-University of Milan and the Department of Psychology of the University of Milan-Bicocca which guaranteed the scientific coordination of the project and the assessment of its efficacy. A pilot program was conducted from 2006 to 2011 involving a small number of cases. In 2011 a service for adolescent and young mothers was created and the PRERAYMI intervention protocol set up (Riva Crugnola et al., 2013b). The project availed itself of the collaboration of George Downing from Pitié-Salpêtrière Hospital in Paris for what concerns that part of the intervention which uses Video Intervention Therapy.

Its principal aim is to improve the mother-infant relationship in the first year of the infant's life, imcreasing maternal responsiveness and reflectivity and mother-infant dyadic emotional regulation, so as to establish secure attachment of the infant to the mother and other attachment figures. It is well known that secure infant attachment is predictive of adequate socio-emotional development and, at the same time, serves a protective function with respect to psychopathological risk in the subsequent stages of development (Sroufe et al., 2005). In the same way maternal sensitivity and the absence of intrusive or withdrawn styles of interaction toward the infant in the first year have a long term impact on the infant's socio-emotional development (Mäntymaa et al., 2004; Lyons-Ruth et al., 2013). One role of particular importance in this regard is played by the styles of emotional regulation which the infant constructs with its caregivers in its first year, as such styles form the basis of infant attachment patterns (Cassidy, 1994; Riva Crugnola et al., 2011).

Its particularity with regard to the other intervention programs is that it systematically uses different methods of intervention in an interdisciplinary manner, integrating the video intervention technique with developmental guidance and psychological counseling. Its principal aim is to support the relationship between the adolescent mother and her child, but also, at the same time, to help the young mother to integrate her experience of maternity with her transition toward adulthood and with her frequent adverse childhood experiences. Compared to other programs, particular attention is also paid to mutual regulation between mother and infant, considered a key aspect of their relationship (Tronick et al., 2005).

The intervention is interdisciplinary and conducted by a team of psychologists, infant neuropsychiatrists and psychomotrists. These therapists all have specific experience within the context of parent and child early infancy prevention programs.

In order to achieve these objectives our intervention is based on three different approaches: video intervention, developmental guidance, and psychological counseling.

For what concerns video intervention the method is the Video Intervention Therapy (VIT; Downing, 2005; Downing et al., 2008), a modified form of cognitive behavioral therapy which also uses psychodynamic elements in its approach (Steele et al., 2014). It has long been used in mental health settings, such as in-patient parent-infant units where a psychiatrically disturbed parent and an infant can be hospitalized together (Downing et al., 2013). A specific protocol is followed for any session (Downing et al., 2013). In the session, the parent's initial observations about the interaction are discussed first. The therapist next highlights a certain number of specific positive events, commenting on what seems positive. Patient and therapist further reflect on these points. The therapist then also points out, in a respectful and supportive manner, a negative interactional pattern. A more extensive therapeutic investigation of this pattern is then undertaken. Through the use of VIT, the therapist can focus on either the “outer movie,” i.e., the objective behavior seen in the video and the “inner movie,” i.e., the thoughts, feelings, and body experience which were present in the mother during the interaction.

The specific aim of using VIT in our program is to analyze micro-analytically, through careful analysis of “micro-details” of the interaction conducted frame by frame by the therapist with the mother, her communication with her infant (Tronick et al., 1998; Beebe et al., 2010). This joint analysis takes place at the level of affective state coordination, supporting mother/infant positive engagement and the ability of the mother to regulate negative emotions (Riva Crugnola et al., 2013b). At the same time there is particular focus on supporting the mother in facilitating her infant's explorative activity and in increasing episodes of joint attention with him/her. Specific importance is also attributed in the intervention to exploring with the mother her feelings upon viewing the video in relation to both her own emotions and those attributed to her infant, the aim being to increase her ability to keep her infant in mind (Slade, 2005). Self-observation of their interaction with the infant by means of the video is a particularly strong stimulus for parents, allowing them in a short space of time to render otherwise unexpressed emotions and representations explicit and to thus activate specific resources (Downing, 2005; Steele et al., 2014). This proves to be particularly useful with adolescent mothers whose emotional awareness and ability to reflect are often limited and still in the course of development. At the same time the possibility of using information relating to the attachment models and reflective capacity of the young mothers drawn from the Adult Attachment Interview conducted with the adolescent mothers at the beginning of the intervention is also of particular importance in the video intervention (Downing, 2005; Moran et al., 2005).

The second aim of the intervention program is to foster in the mother the process of integrating her experience of maternity and her relationship with the infant with her transition toward adulthood. In those most at risk cases an adolescent mother may distance herself from her experience of maternity, perceiving it as extraneous to her existential and developmental condition, delegating care of the infant to others both physically and emotionally, with the infant being abandoned in the most problematic cases. This may be due to the fact that many adolescent mothers have had negative or traumatic experiences with their parents and caregivers which are reactivated by having to deal with their small infants and the intense emotions the infants suscitate in them. Just a few sessions of psychological counseling are held in less problematic cases and these are aimed at facilitating the mother's transition toward adulthood. However, counseling lasts longer and may be extended to the entire period of the intervention in cases (around 50%) in which the mothers have had traumatic experiences or are currently having problems with regard to their relationship with their infant, their family of origin or their partner. In such cases the mother is helped to reflect in depth upon conflictual aspects of her past and/or current relationships with her own parents, and upon how these now reflect her relationship with her infant (Lieberman and Pawl, 1993). The mother's elaboration of trauma is an important feature of the intervention, also in the light of studies which have shown that researchers have had difficulties in intervening, through attachment-based interventions, with adolescent mothers who are disorganized with respect to abuse they have suffered (Moran et al., 2005; Berthelot et al., 2015).

A third level of intervention provides mothers with developmental guidance (Papoušek et al., 2008). In monthly sessions the stages of development of the infant and its rhythms of regulation, which mothers are often little aware of, are illustrated. The meetings are led by a psychomotrist who focuses on developmental guidance (Papoušek et al., 2008). In this context the infant's motor and cognitive development is monitored using the Bayley Scales (2005) at 6 and 10 months. The results are discussed in specific meetings led by an infant neuropsychiatrist and a psychomotrist with the mothers and fathers in order to identify with them both the developing skills of the infant and any problems.

#### The intervention protocol

The intervention takes place in a specially dedicated outpatient unit of the hospital. The aim is to provide young mothers with a protected area which they can inhabit as if it were their own. Adolescent mothers often find themselves living with their family of origin or with their partner's family in crowded conditions which offer them little chance to protect their individual relationship with the infant. Moreover, in order to ensure that a relationship between the young mother and the service is created which will act as a secure basis for her budding relationship with her infant, from the beginning to the end of the intervention the mothers are always followed by the same two therapists—a psychologist and a psychomotrist—during the meetings in order to ensure that they have a sense that the intervention is continuous.

Intervention starts when the infant is 2 months old and concludes at 9 months. There are around 15 meetings in all. The program begins with an initial meeting at infant 2 months with the mother. During this meeting an anamnestic form is compiled with the mother and risk and protection factors (socio-economic condition, relationship with the infant's father, social support, educational level, progression of pregnancy, and adverse experiences) with respect to her relationship with the baby are thus identified.

At infant 3 months, self-report questionnaires are used to assess levels of parental stress (PSI-SF; Abidin, 1995) and post-partum depression (PDSS; Beck and Gable, 2002). The “state of mind of the mother” with respect to attachment is also examined using the *Adult Attachment Interview* (Main et al., 2002).

When possible, infants' fathers are also included in the intervention protocol. They are, however, often unavailable because of work commitments or current lack of engagement. Nonetheless, in order to foster collaboration between mothers and fathers, the more informative meetings which focus on developmental guidance are conducted at times which are compatible with the fathers' working hours. In the course of the intervention we are also in contact with the parents of the adolescent mothers, albeit not systematically.

Video intervention begins at 3 months and is conducted monthly until infant 9 months (6 sessions). Meetings are led by a psychologist and a psychomotrist using the VIT method. During each meeting the mother and the infant are videorecorded for 5–10 min in free-play situations with suitable toys being available. In the following meeting, which occurs a few days after that of the videorecording, the recording is discussed with the mother.

The mothers are also given counseling sessions conducted by psychologists (an average of 6 sessions). As we stated above the aims of the sessions are to foster integration of their experience of becoming a mother with their transition toward adulthood or, in some more problematic cases, to tackle issues linked to their past or to difficult present relationships, such as with the partner or family of origin.

The developmental guidance is conducted monthly by a psychomotrist (an average of 6 sessions) as above.

There are two follow-ups after the conclusion of the intervention for the purposes of assessing its efficacy. The first takes place at infant 14 months in order to evaluate the type of attachment developed by the infant to the mother assessed using the *Strange Situation Procedure* (Ainsworth et al., 1978). The second follow-up is carried out at infant 24 months when mothers are given the *Child Behavior CheckList* (CBCL; Achenbach and Rescorla, 2001) in order to evaluate the efficacy of the intervention with regard to the infant's psycho-pathological risk.

### Research aims

For the purposes of assessing the effectiveness of the PRERAYMI intervention program a preliminary study was conducted which examined the effect of the intervention at an intermediate stage, i.e., 3 months after the start of the video intervention and in the final stage at 9 months. The aim of the study was to assess changes in interaction styles and dyadic emotion regulation after 3 and 6 months of intervention in the adolescent mother-infant dyads who had used the intervention, comparing them with a control group made up of dyads which had not used the intervention. In relation to this aim we also asked ourselves, at an exploratory level, whether the attachment models and reflective capacity of the mothers assessed at the start of the intervention could have a moderating effect on its efficacy.

We formulated the following hypotheses with respect to these aims: (a) the adolescent mothers who use the intervention will increase their Sensitivity style and decrease their Controlling style and their infants will have more Cooperative and fewer Passive styles after 3 and 6 months of intervention compared to mothers and infants in the control group; (b) the dyads with adolescent mothers who use the intervention (vs. control group) will display an increase in the amount of time spent in match states and in positive match states, a decrease in the amount of time spent in negative match states and mismatch and a greater capacity to move from mismatch to match states (repair) compared to the dyads of the control group after 3 and 6 months of intervention; (c) the dyads with intervention will increase the amount of time spent in play with objects after 3 and 6 months compared to the dyads of the control group.

## Materials and methods

### Design

The dyads were recruited at the Obstetrics and Gynaecology Department of the San Paolo Hospital of Milan and at Family Counseling Services in the Province of Milan. A total of 48 adolescent mothers and their infants were enrolled in the pilot study. 32 dyads were assigned to the intervention group and 16 dyads were assigned to the treatment-as-usual control group. Inclusion criteria included: able to speak and understand the Italian language; 14–21 years old; and having a first child. The study protocol was approved by the institutional review board of the San Paolo Hospital of Milan. All subjects gave written informed consent in accordance with the Declaration of Helsinki.

### Participants

In the adolescent mother-infant dyads of the intervention group, mothers had a mean age of 18.75 (*SD* = 1.43), SES was medium in 15% of cases and low in the remaining 85%, and 71% had left school at the age of 16. 72% lived with their parents and 28% lived with their partners. Sixty-five percent of the infants were the result of unwanted pregnancies, 90% of the adolescents had mothers who had also had early pregnancies and 46% had a history of abuse or neglect. In the control group, mothers had a mean age of 17.94 (*SD* = 1.94), SES was high in 15% of cases and low in the remaining 85%, and 85% had left school at the age of 16. 50% lived with their parents and 50% lived with their partners. Seventy-five percent of the infants were the result of unwanted pregnancies, 87% of the mothers had mothers who had also had early pregnancies, and 50% had a history of abuse or neglect. In both groups, the infants were all born full term, without organic pathologies.

Most of the adolescent mothers of both groups were Italian. The rest were European or Latin American who knew the Italian language and were integrated into the Italian cultural context. In the intervention group 26 mothers were Italian, 2 European and 4 Latin American and in the control group 14 were Italian and 2 Latin American.

At infant 9 months, the number of participants was much smaller: 18 in the intervention group and 10 in the control group because some dyads dropped out of the program after 3 months of treatment (*n* = 16) or the infant was not 9-months old yet (*n* = 4) at the time of the post-intervention assessment.

### Procedure and program implementation

At infant 3, 6, and 9 months, mother-infant interactions were video-recorded and coded with the Care-Index (Crittenden, 1998) and a modified version of Infant and Caregiver Engagement Phases (Weinberg and Tronick, 1999; Riva Crugnola et al., 2013a) in order to evaluate the changes in interactions and emotion regulation of the mother and infant after 3 and 6 months in the intervention group and in the control group.

The Adult Attachment Interview (George et al., 1985) was also administered to the mothers at infant 3 months to evaluate maternal attachment representations and reflective functioning (Fonagy et al., 1998).

As previously described in the intervention group intervention began at 3 months (risk having been identified at 2 months) with video intervention sessions every month. The mean number of video intervention sessions was 2.3 after 3 months of treatment and 4.1 after 6 months of treatment.

Counseling sessions were also conducted each month. In the intervention group the mean number of counseling sessions was 3.5 after 3 months of intervention and 5.5 after 6 months of intervention.

Developmental guidance sessions were also conducted monthly. In the intervention group the mean number of developmental guidance sessions was 3.7 after 3 months of intervention and 5.2 after 6 months of intervention.

The control group did not follow the intervention program, but did receive routine postnatal well-woman health visits and well-baby healthcare visits.

### Measures

#### Adult attachment interview (AAI; George et al., 1985)

The AAI is a semi-structured interview which explores the interviewees' relations with their parents as children, including early separation and means of comfort-seeking. According to the Main coding system (Main et al., 2002), based on 9-point scales, each interview was assessed for the following categories: Secure/Autonomous (F), Dismissing (Ds), Preoccupied (E), Unresolved/Disorganized (U). The interviews assigned to the U category received a secondary score of Secure/Autonomous, Dismissing or Preoccupied. According to this system, autonomous secure attachment involves consistent and objective narration of attachment experiences and their assessment; dismissing attachment involves inconsistent narration of attachment experiences with idealization of attachment figures, distinguished by generally positive descriptions of the latter which are not supported and/or are contradicted by specific episodes, difficulty in remembering and underestimation of these experiences; preoccupied attachment involves inconsistent narration characterized by vagueness and prolixity together with worry and/or anger being expressed toward attachment figures; unresolved/disorganized attachment involves failure to process traumatic episodes (maltreatment, abuse, etc.) and mourning; lastly unclassifiable attachment involves the co-presence of contradictory mental states with regard to attachment.

The interviews were scored by the first author, who is trained and reliable with the AAI coding system. The second judge, also trained and reliable with the AAI coding system, rated 20% of the interviews. Concordance between the two coders for the four way classifications was 85% (*k* = 0.70) and for the two way classifications (secure vs. insecure) 100% (*k* = 1.00).

#### Reflective functioning scales

The reflective functioning scale (*Reflective Functioning, RF*; Fonagy et al., 1998) applied to the Adult Attachment Interview allows assessment of the mentalization of the interviewee, understood as the capacity to give meaning to one's own and others' experiences in terms of mental states and emotions. Reflective function is assessed by means of a scale from −1 to 9. The category *Negative RF (-1)* covers interviewees who are confused or hostile and refuse all attempts on the part of the interviewer to get them to begin any reflection; the category *Lacking in RF (1)* covers interviewees in whom the reflective function is totally or almost totally absent. They may mention mental states occasionally with respect to themselves or others, but such mentioning is not connected to feelings underlying the behavior of the interviewee; the category *Questionable or Low RF (3)* covers interviewees who display some evidence of awareness of mental states, albeit at a fairly rudimentary level. The category *Ordinary RF (5)*covers interviewees who possess some type of model of the mind of attachment figures and of their own mind which is relatively consistent if simple; the category *Marked RF (7)* covers interviewees who demonstrate awareness of the nature of mental states for the entire interview and express efforts to reflect on the mental states underlying behavior; the category *Exceptional RF (9)* covers interviewees who are exceptionally sophisticated and surprising, adopting causal reasoning in which mental states are used. Reliability between coders was calculated on 20% of the interviews through the intraclass correlation coefficient and was ICC = 0.82.

Both coders (the first and second authors) were trained and reliable for the RF scales.

#### Care-index

The Child-Adult Relationship Experimental Index (Care-Index; Crittenden, 1998) is a coding instrument for caregiver-infant interaction from 0 to 15 months. There are three scales which measure the behavior of the adult: *Sensitivity*, covers responsive behavior, involvement which is positive and in harmony with the emotions of the infant and his activities; *Controlling*, covers directive behavior characterized by open or implicit hostility (pseudo-sensitive) and interference with the activity of the infant, such as excessive handling of the infant's body, raised tone of voice and hyperstimulation; *Unresponsiveness*, covers behavior marked by physical and emotional detachment from the infant, such as silence, failure to offer play, little or no involvement. The infant's behavior is assessed according to four scales: *Cooperative*, covers behavior associated with the expression of positive emotions, centered on undertaking actions and accepting those offered by the caregiver; *Compliant-Compulsive*, covers cautious and inhibited behavior with an indirect and compliant approach toward the mother; *Difficult*, covers behavior which is explicitly resistant to proposals of the mother, such as avoiding gaze, crying, throwing objects and negative vocalization; *Passive*, covers behavior aimed at reducing physical and emotional contact with the mother, such as failure to vocalize and looking at surroundings. For all scales the scores vary from 0 to 14. With respect to the scores given to maternal sensitivity and infant cooperativeness, the range of scores 0–4 is considered high risk and indicates poor sensitivity of a problematic type requiring therapeutic intervention, the range of scores 5–6 is the range within which intervention is considered necessary as maternal sensitivity is only marginally adequate, 7–10 indicates adequate sensitivity and 11–14 indicates very good sensitivity.

Reliability between observers was calculated on 20% of the observations of the dyads through the intraclass correlation coefficient and was ICC = 0.88 for maternal behavior and ICC = 0.83 for infant behavior. The two coders were blind to the classification of maternal attachment and reflective function scores.

#### Infant and caregiver engagement phases

The interactions were also coded by the Infant Caregiver and Engagement Phases (ICEP; Weinberg and Tronick, 1999), which we modified to analyze the interaction between mother and infant concerning objects (Riva Crugnola et al., 2013a). This is a system which evaluates the behavior of mother and infant during face-to-face play on the basis of emotions expressed, gaze direction, vocalization, and verbalization. Since the original coding system was created to evaluate mother and infant interaction in the Still Face paradigm, which does not involve the use of objects, we introduced new categories with the aim of exploring the way in which infants and their mothers direct attention to objects during play. These categories differentiate between (1) the infant's attention to objects offered by his/her mother, or chosen by him/herself and (2) the mother's involvement with an object chosen by the infant, or an object chosen by the mother, as shown in Table 1.

**Table 1:** **Infant and mother behavioral codes**.

Infant codes	Negative engagement	Infant is negative, protesting with facial expressions of anger, annoyance, often with crying or withdrawn/passive and minimally engaged with the mother and the environment.
	Social positive engagement	Infant is displaying facial expressions of joy, astonishment and smiles. SPE is considered play with or without objects, but social play.
	Orientation to objects offered by the mother^*^	Infant is looking, touching, playing with objects offered by the mother.
	Orientation to objects not offered by the mother^*^	Infant is looking, touching, playing with objects not offered by the mother.
	Orientation to environment	Infant is visually exploring the setting without focalizing attention on any specific object.
	Social monitor	Infant's attention is directed toward mother's face. He/she is looking at her. ward mother's face. He/she is looking at her.
	Allows comforting^*^	Infant lets the mother comfort him/her when he/she is upset (e.g., she rocks the infant).
	Allows caretaking^*^	Infant lets the mother provide caregiving (position him, blow his/her nose).
	Unscorable	Infant's face is obscured (e.g., his/her face is covered by the mother's body or is outside the view of the camera) or the infant is asleep.
Mother codes	Negative engagement	Mother is negative, intrusive toward the infant's physical space, activities and objects, hostile or withdrawn (minimally engaged with the infant's activities).
	Social positive engagement	Mother is interacting with the infant through facial expressions of joy and interest, with positive vocalizations, motherese and social play.
	Involvement in play^*^	Mother joins in the game with the object chosen by the infant.
	Offer of object^*^	Mother is offering a new object chosen by her to the infant.
	Social monitor	Mother is looking at the infant and his/her activities.
	Comforting^*^	Mother is comforting the infant when he/she is upset (e.g., he/she cries).
	Caretaking^*^	Mother is caregiving the infant, positioning him, blowing his/her nose.
	Non-infant focused	Mother is not attending to the infant or to the infant's activities.
	Call for infant's attention^*^	Mother is trying to draw the infant's attention to her or to an object (e.g., calling the infant, shaking the object, making noises).
	Unscorable	Mother's face is obscured (e.g., her face is covered).

*This coding system is an elaboration of ICEP (Weinberg and Tronick, 1999). Categories with an asterisk were not provided in the ICEP, being introduced for the purposes of our studies (Riva Crugnola et al., 2013a)*.

Maternal and infant behaviors were analyzed second by second, using the Noldus Observer XT system. Coding was continuous and occurred for every instance of a behavior. Maternal and infant behaviors were analyzed second by second. The codes were mutually exclusive. Infant and maternal behavior was coded separately, and at different times, by the same researcher. It was decided to use the same coder given the interactive characteristic of many codes (e.g., those regarding play with objects). It was therefore important that in coding one member of the dyad the researcher also bore in mind the behavior of the other.

In order to evaluate matched and mismatched states, we combined the second-by-second codes according to the “Global States” they represented, using three categories: neutral, positive, and negative, via the GSEQ program (Bakeman and Quera, 1995) as presented in Table 2. The Sleeps, Observes Stranger and Unscorable categories of the infant and the Unscorable category of the mother were not included in the behavior analysis or in the grouping of affective states since they were low frequency and not relevant for the assessment of individual and dyadic emotional regulation.

**Table 2:** **Definition of affective states: positive, neutral, negative**.

**Affective states**	**Codes**
Infant positive	Social positive engagement, orientation to objects not offered by the mother, orientation to objects offered by the mother
Infant neutral	Social monitor, orientation to the environment, allows caretaking, allows comforting
Infant negative	Negative engagement
Mother positive	Social positive engagement, offer of object, involvement in play
Mother neutral	Social monitor, call for infant's attention, non-infant focused, caretaking, comfort
Mother negative	Negative engagement

The concept of match and mismatch used by Tronick et al. (2005) was adopted to investigate the capacity to coordinate affective states at dyadic level of mother and infant. Coordinated affective states are defined as “match” and correspond to moments in which mother and infant share and express the same affective states at the same time, whether they be positive, negative or neutral. Non-coordinated affective states are defined as “mismatch” and correspond to moments in which mother and infant have a different affective state at the same time. We then calculated the relative duration of different coordinated affective states (positive match, negative match, neutral match) and of non-coordinated affective states (mismatch; Infant positive/Mother negative, Infant positive/Mother neutral, Infant negative/Mother positive, Infant negative/Mother neutral, Infant neutral/Mother positive, Infant neutral/Mother negative). In calculating total matches we considered the sum of the duration of Infant positive/Mother positive and Infant neutral/Mother neutral matches. The Infant negative/Mother negative was not, however, included because it could not be considered an adequate state of affective coordination (Reck et al., 2011). Total mismatches correspond to the sum of all six different states of mismatch (see Table 2). Lastly, we also calculated repair (Tronick et al., 2005), i.e., the dyadic capacity for repair of mismatches according to frequency per minute of passage from mismatch to positive or neutral match (see Table 2).

The coder evaluated the behavior of both mother and infant. A second coder operating independently of the first also coded the behavior of the mothers and infants of 20% of the dyads. Inter-rater agreement in the second-by-second codes calculated by Cohen's Kappa coefficient (Cohen, 1960) was 0.89 for the observation of maternal behavior and 0.88 for the observation of infant behavior. The two coders were blind to the classification of maternal attachment and the scores of reflective function.

### Data analysis

The SPSS Statistic 21 package was used for all analyses. Descriptive statistics and comparisons were calculated between intervention and control groups with respect to demographic characteristics and baseline measures to determine the equivalence of the two groups; *t*-tests for the continuous variables and Chi-square test (or Fisher's exact tests) for nominal variables were applied.

To evaluate the effects of intervention on mother-infant interaction, Generalized Linear Mixed Models (GLMMs) with fixed effects including group, time and interaction between group and time and subject level random intercepts were used to analyze group differences and changes in mother-infant interaction from 3 to 9 months between the two different groups. In particular, the effect of interaction between group and time was used to evaluate the effectiveness of the intervention, i.e., whether the intervention group improved more than the control group over time. GLMM procedure for correlations between repeated measures within subjects allows analyzing both fixed and time-varying covariates and automatically handles missing data.

Maternal attachment representations (secure vs. insecure) were used as an additional factor which was tested in separate GLMMs. It was decided to use only maternal attachment as a possible moderating factor on the effectiveness of the intervention, given that there was a high multicollinearity relationship between maternal attachment and scores of reflective functioning.

## Results

### Socio-demographic characteristics

Table 3 shows the comparison made of the intervention and control groups with respect to socio-demographic variables and risk factors with the Chi-square test (or Fisher's exact test) and the *t*-test according to whether the variables were nominal or continuous in order to determine the equivalence of the two groups. The results did not indicate any significant differences between the intervention and control groups at the baseline stage of infant 3 months.

**Table 3:** **Socio-demographic characteristics**.

	**Intervention group (*N* = 32)**	**Control group (*N* = 16)**	**Intervention vs. control**
**MOTHER**			
Age Mean (SD; range)	18.75 (1.43; 15–21)	17.94 (1.94; 15–21)	ns
**Marital Stastus**			
Single	21 (65%)	14 (87%)	ns
Married	11 (35%)	2 (13%)	
**Living Arrangements**			
With a partner	9 (28%)	8 (50%)	ns
With a parents	23 (72%)	8 (50%)	
**Education**			
No school degree	0 (0%)	1 (6%)	
Less than secondary education	25 (78%)	14 (88%)	ns
Higher degree	7 (22%)	1 (6%)	
SES (Mean; SD)	19.79 (5.36)	21 (5.99)	ns
History of parenthood at a young age	29 (90%)	14 (87%)	ns
Trauma	15 (46%)	8 (50%)	ns
Unwanted pregnancy	21 (65%)	12 (75%)	ns
**INFANT**			
**Sex**			
Female	20 (62%)	10 (62%)	ns
Male	12 (38%)	6 (38%)	

*Number of subjects (N), standard deviation (SD), level of significance (p), and non- significance (ns)*.

### Maternal representation of attachment and reflective functioning

In the intervention group, 12 adolescent mothers had secure attachment and 20 mothers insecure attachment of whom 8 Dismissing, 5 Preoccupied, 5 Unresolved/Disorganized, and 2 Cannot Classified; 3 mothers of the control group had secure attachment and 10 insecure attachment of whom 3 Dismissing, 4 Preoccupied and 3 Unresolved/Disorganized. Three mothers (18.7%) of the control group did not take part in the interview.

In both intervention and control groups, 60% of adolescent mothers had an insecure attachment model, with a distribution similar to that of clinical and at risk samples (Bakermans-Kranenburg and van IJzendoorn, 2009). The distribution of attachment groups did not significantly differ between the intervention and control groups (Fisher's exact test = 0.86; *ns*).

In both groups the adolescent mothers also had a low score in reflective functioning. In particular the mothers of the intervention group had an average score of 2.84 and the mothers of the control group an average of 2.23. The difference in the averages of scores on reflective functioning between the two groups was not significant [*t*_(43)_ = 1.08, *ns*].

### Mother-infant interaction

Interaction styles at 3 months, at the baseline pre-intervention stage, were analyzed first of all to see whether there were any differences between intervention and control groups. Analysis conducted with the *t*-test did not indicate any significant differences between the two groups at 3 months with regard to either mother or infant styles. At the pre-intervention assessment, following the Care-Index coding scheme, the average scores in the range of sensitivity of the dyads was 5 for the mothers of both groups and 3.2 for the infants of the control group and 4.3 for the infants of the intervention group. This band is considered by Crittenden (1998) to be at risk and in need of “future intervention.”

**Figure 1:**
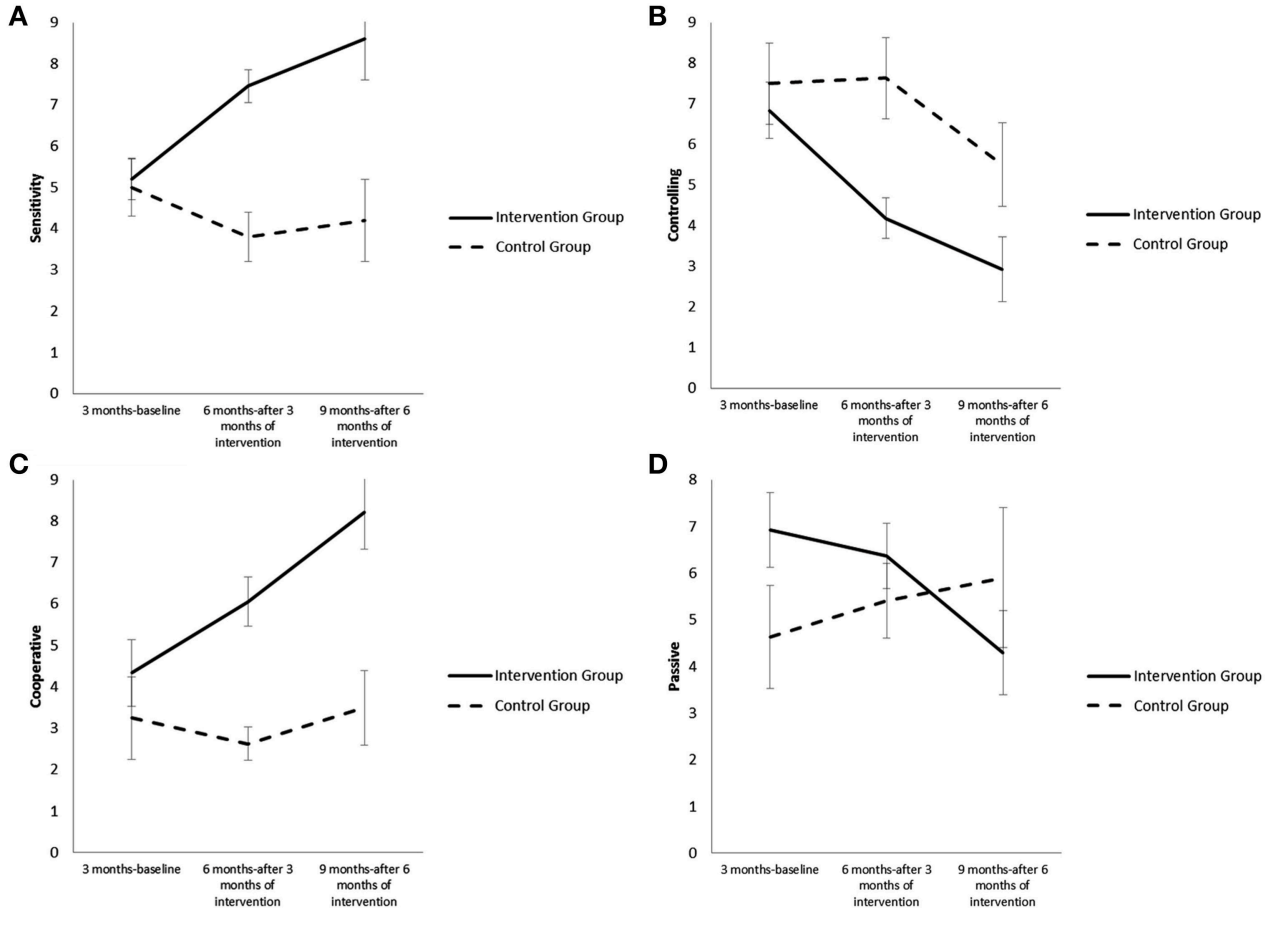
**(A)** Differences in Sensitivity style for the mothers who participated in the intervention and for the mothers of the control group from 3 to 9 months. **(B)** Differences in Control style for the mothers who participated in the intervention and for the mothers of the control group from 3 to 9 months. **(C)** Differences in Cooperative style for the infants who participated in the intervention and for the infants of the control group from 3 to 9 months. **(D)** Differences in Passive style for the infants who participated in the intervention and for the infants of the control group from 3 to 9 months.

Analysis with the Generalized Linear Mixed Models indicated that there was a significant main effect of group [*F*_(1, 46)_ = 14.08, *p* = 0.000] and a significant interaction effect of group × time for the Sensitivity style of the mother [*F*_(2, 74)_ = 10.87, *p* = 0.000; see Figure 1A]. The adolescent mothers of the intervention group showed an increase in Sensitivity style compared to the control group after both 3 months of intervention [*b* = 3.43, *t*_(70)_ = 3.83, *p* = 0.000] and 6 months of intervention [*b* = 4.31, *t*_(76)_ = 4.01, *p*= 0.000]. Furthermore, the effect of intervention was greater from 3 to 6 months than from 6 to 9 months [*b* = 0.88, *t*_(76)_ = 0.82, *ns*].

There were also significant main effects of group [*F*_(1, 47)_ = 8.75, *p* = 0.005] and time [*F*_(2, 75)_ = 10.42, *p* = 0.000] and an interaction effect of group × time for Controlling style [*F*_(2, 75)_ = 4.44, *p* = 0.015]. The adolescent mothers of the intervention group compared to the mothers of the control group showed a greater decrease in Controlling style after both 3 months of intervention [*b* = −2.78, *t*_(71)_ = −2.80, *p* = 0.006] and 6 months of intervention [*b* = −2.46, *t*_(77)_ = −2.07, *p* = 0.041]. Furthermore, the effect of intervention was greater from 3 to 6 months than from 6 to 9 months [*b* = 0.31, *t*_(77)_ = 0.26, *ns;* see Figure 1B]. However, the mothers of the control group differed in that they showed a decrease in Sensitivity style and maintained a high Controlling style from 3 to 9 months. There were no significant main effects of group [*F*_(1, 48)_ = 0.85, *ns*] and time [*F*_(2, 77)_ = 3.10, *ns*] or interaction effect [*F*_(2, 77)_ = 3.06, *ns*] for the Unresponsiveness style of the mother.

Analysis also indicated that there were significant main effects of group [*F*_(1, 46)_ = 15.32, *p* = 0.000] and time [*F*_(2, 75)_ = 5.28, *p* = 0.007] and a significant interaction effect of group × time for the Cooperative style of the infant [*F*_(2, 75)_ = 4.54, *p* = 0.014]. The infants of the intervention group compared to the infants of the control group had an increase in scores of the Cooperative style after both 3 months of intervention [*b* = 2.34, *t*_(71)_ = 2.17, *p* = 0.033] and 6 months of intervention [*b* = 3.60, *t*_(78)_ = 2.80, *p* = 0.006]. Furthermore, the effect was greater from 3 to 6 months than from 6 to 9 months [*b* = 1.26, *t*_(78)_ = 0.98, *ns;* see Figure 1C]. There was also a significant interaction effect of group × time for the Passive style of the infant [*F*_(2, 77)_ = 3.14, *p* = 0.049] and there was no significant main effect of group [*F*_(1, 47)_ = 0.37, *ns*] and time [*F*_(2, 77)_ = 0.55, *ns*]. The infants of the intervention group had a decrease in scores in Passive style at 9 months after 6 months of intervention compared to the infants of the control group [*b* = −3.95, *t*_(80)_ = −2.50, *p* = 0.014] while the effect was not yet significant at 6 months after 3 months of intervention [*b* = −1.37, *t*_(72)_ = −1.03, *ns;* see Figure 1D]. The infants of the control group differed in that Cooperative style at 9 months did not change with respect to 3 months, and there was a decrease at 6 months, and Passive style increased from 3 to 9 months. There was, however, no significant main effect of time [*F*_(2, 73)_ = 1.23, *ns*] or interaction effect [*F*_(2, 73)_ = 1.64, *ns*] for Compliant-Compulsive style and there were no significant main effects of group [*F*_(1, 48)_ = 3.86, *ns*] and time [*F*_(2, 77)_ = 0.49, *ns*] or interaction effect [*F*_(2, 77)_ = 0.96, *ns*] for infant Difficult style.

At the post-intervention assessment by the Care-Index, the intervention group at 9 months reached an average score of 8.6 for mothers and 8.2 for infants, which according to Crittenden (1998) indicates an adequate quality of mother and infant interaction, while the control group went down to an average score of 4.0 for the mothers and 3.5 for the infants, remaining in the “in need of further intervention” category.

### Play with objects and affective states coordination

Firstly, we analyzed the individual behaviors of mother and infant in relation to play with objects and affective coordination and repair assessed with ICEP at 3 months at the pre-intervention baseline stage in order to see whether there were any differences between intervention and control groups. Analysis conducted with the *t*-test did not indicate any significant differences between the two groups at 3 months.

We present hereunder the results for the individual categories of behavior as per ICEP concerning play with objects. For the other categories we present the results for dyadic affective coordination, basing ourselves on the grouping of the categories into global affective states (positive, negative, and neutral).

**Figure 2:**
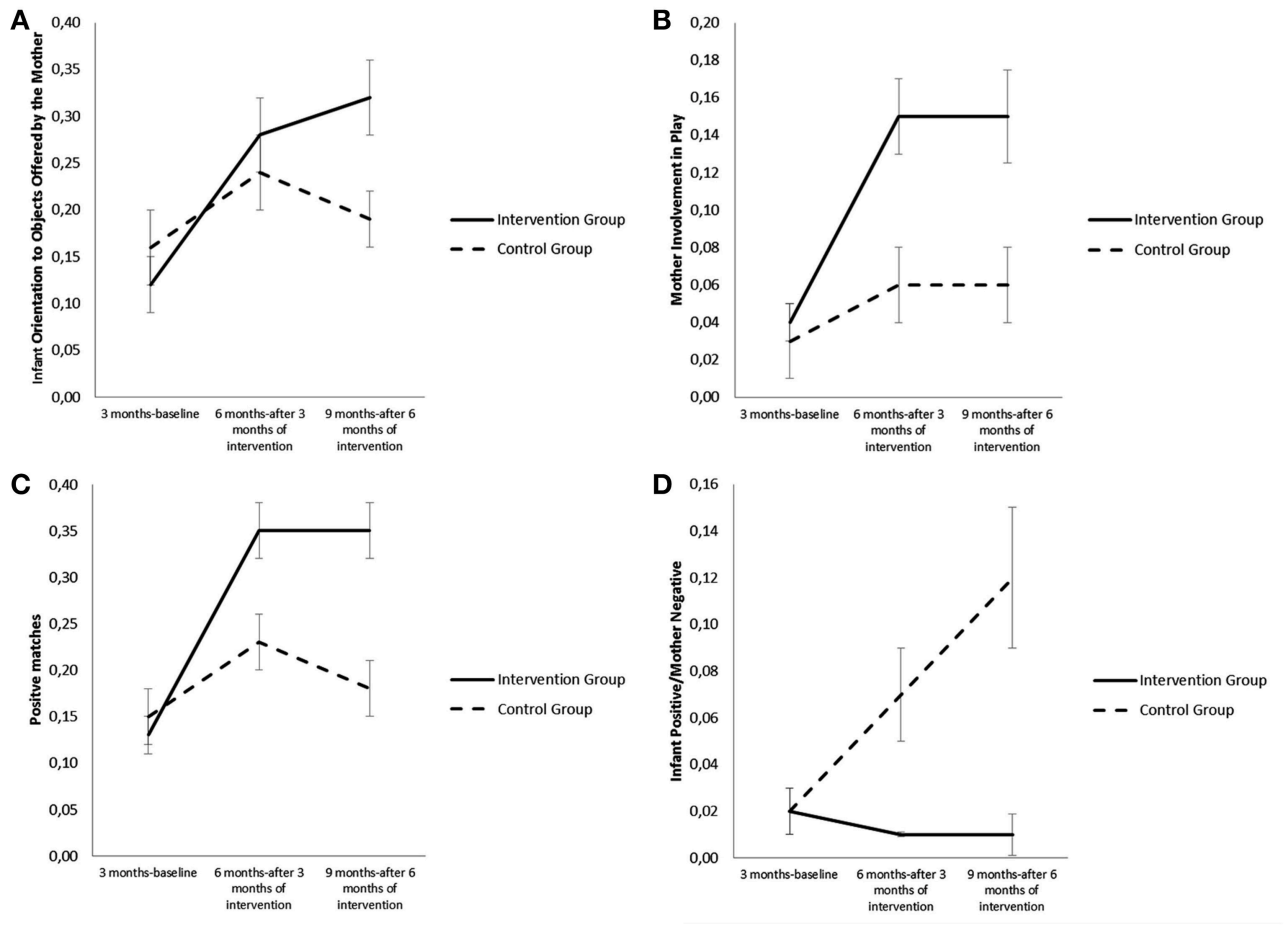
**(A)** Differences in Infant Orientation to Objects Offered by Mother for the infants who participated in the intervention and for the infants of the control group from 3 to 9 months. **(B)** Differences in Mother Involvement in Play for the mothers who participated in the intervention and for the mothers of the control group from 3 to 9 months. **(C)** Differences in Positive Matches for the dyads who participated in the intervention and for the dyads of the control group from 3 to 9 months. **(D)**. Differences in Infant Positive/Mother Negative Mismatches for the dyads who participated in the intervention and for the dyads of the control group from 3 to 9 months.

Analysis with the Generalized Linear Mixed Models indicated that there was a significant main effect of time [*F*_(2, 84)_ = 9.16, *p* = 0.000] and a significant interaction effect of group × time for “Orientation to Object Offered by the Mother” behavior of the infant [*F*_(2, 84)_ = 3.23, *p* = 0.044]. At 9 months compared to 3 months, the infants of the intervention group showed a greater increase than did the infants of the control group in the amount of time spent in “Orientation to Object Offered by the Mother” behavior [*b* = 0.17, *t*_(90)_ = 2.50, *p* = 0.014], while there was not as yet any significant difference at 6 months [*b* = 0.08, *t*_(76)_ = 1.45, *ns;* see Figure 2A]. There was also a significant main effect of time for “Orientation to Objects not offered by the Mother” behavior of the infant [*F*_(2, 78)_ = 42.75, *p* =0.000] which indicated an increase from 3 to 9 months in both groups. However, the main effect of group [*F*_(1, 46)_ = 1.44, *ns*] and the interaction effect of group × time [*F*_(2, 78)_ = 2.22, *ns*] for this behavior were not significant.

With respect to maternal behavior of play with objects, there were significant main effects of group [*F*_(1, 46)_ = 10.14, *p* = 0.003] and time [*F*_(2, 76)_ = 11.98, *p* = 0.000], and a significant interaction effect of group × time for the maternal behavior of “Involvement in Play” [*F*_(2, 69)_ = 5.18, *p* = 0.008]. The adolescent mothers of the intervention group compared to the mothers of the control group had a greater increase in the amount of time spent in “Involvement in Play” behavior after both 3 months of intervention [*b* = 0.09, *t*_(70)_ = 3.04, *p* = 0.003] and 6 months of intervention [*b* = 0.08, *t*_(81)_ = 2.24, *p* = 0.027; see Figure 2B]. Furthermore, the effect was greater from 3 to 6 months than from 6 to 9 months [*b* = 0.01, *t*_(80)_ = 0.32, *ns*]. There were no significant main effects of group [*F*_(1, 45)_ = 0.03, *ns*] and time [*F*_(2, 80)_ = 1.88, *ns*] or interaction effect [*F*_(2, 80)_ = 0.97, *ns*] for the mother's behavior of “Offer of Object.”

For what concerns affective state coordination, the results showed that there were significant main effects of group [*F*_(1, 44)_ = 10.28, *p* = 0.002] and time [*F*_(2, 78)_ = 14.32, *p* = 0.000] and a significant interaction effect of group × time for positive matches [*F*_(2, 78)_ = 4.82, *p* = 0.011]. The dyads of the intervention group compared to the dyads of the control group showed a greater increase in the amount of time spent in states of positive match after both 3 months of intervention [*b* = 0.14, *t*_(71)_ = 2.55, *p* = 0.013] and 6 months [*b* = 0.17, *t*_(84)_ = 2.69, *p* = 0.008]. Furthermore, the effect was greater from 3 to 6 months than from 6 to 9 months [*b* = 0.03, *t*_(84)_ = 0.50, *ns;* see Figure 2C].

However, there were no significant interaction effects on negative matches [*F*_(2, 84)_ = 0.93, *p* = *ns*] and neutral matches [*F*_(2, 76)_ = 0.15, *ns*]. There was, however, a significant main effect of time for neutral matches [*F*_(2, 76)_ = 19.42, *p* = 0.000] which indicated a decrease from 3 to 9 months in both groups.

Compared to individual mismatches, there were significant main effects of time [*F*_(2, 85)_ = 4.23, *p* = 0.018] and group [*F*_(1, 53)_ = 16.66, *p* = 0.000] and a significant interaction effect of group × time [*F*_(2, 85)_ = 6.69, *p* = 0.002] for the mismatch “Infant positive/Mother negative.” In the dyads of the intervention group compared to the dyads of the control group there was a decrease in the amount of time spent in mismatch “Infant positive/Mother negative” after both 3 months of intervention [*b* = −0.60, *t*_(79)_ = −2.45, *p* = 0.016] and 6 months[*b* = −0.10, *t*_(89)_ = −3.50, *p* = 0.001]. Furthermore, the effect was greater from 3 to 6 months than from 6 to 9 months [*b* = −0.04, *t*_(89)_ = −0.1.40, *ns*]. However, in the dyads of the control group there was an increase in the mismatch “Infant positive/Mother negative” from 3 to 9 months (see Figure 2D). Lastly, there were no significant interaction effects for the other individual mismatches.

**Figure 3:**
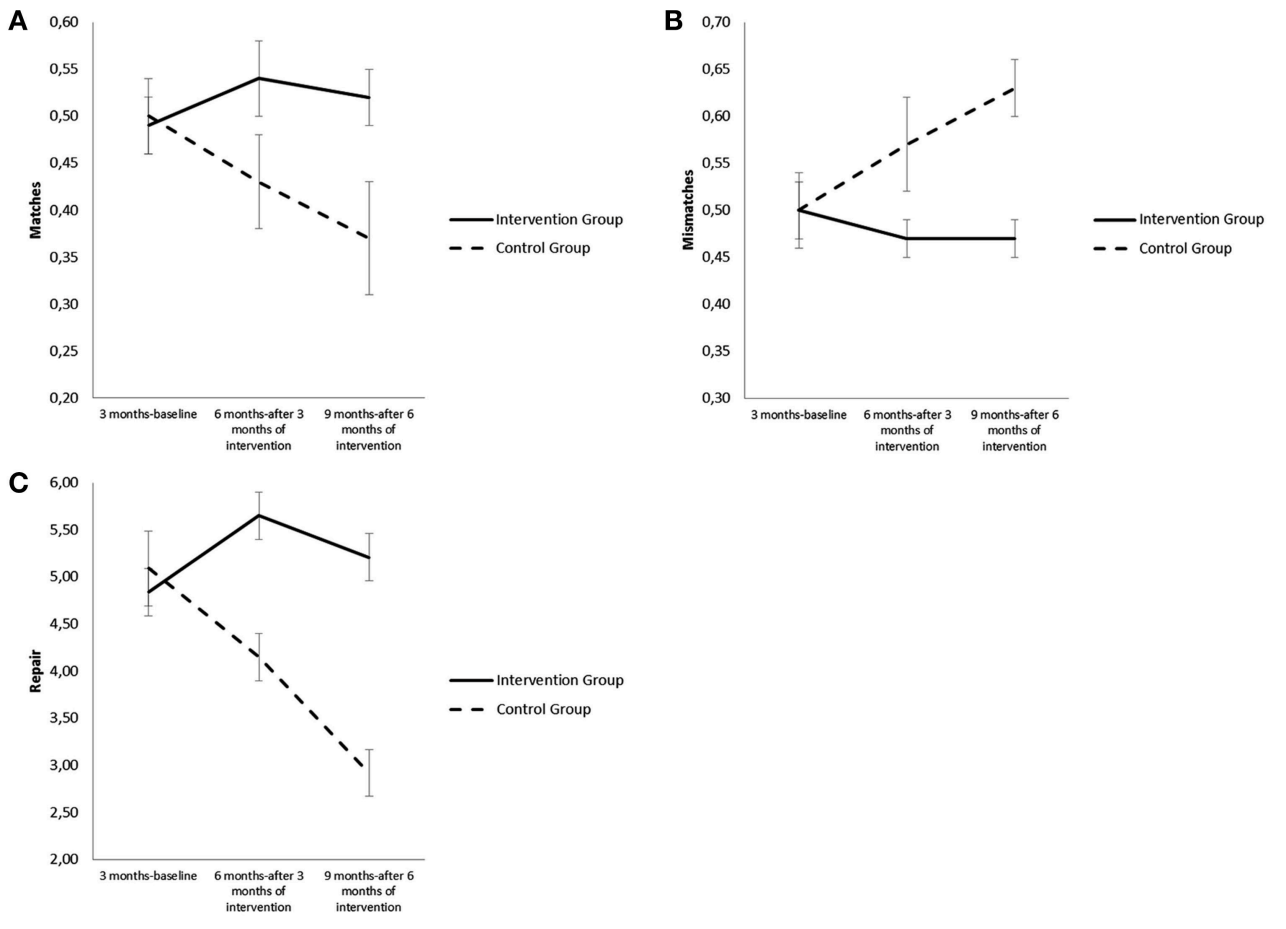
**(A)** Differences in Matches, for the dyads who participated in the intervention and for the dyads of the control group from 3 to 9 months. **(B)** Differences in Mismatches for the dyads who participated in the intervention and for the dyads of the control group from 3 to 9 months. **(C)** Differences in Repair for the dyads who participated in the intervention and for the dyads of the control group from 3 to 9 months.

Analysis also indicated that there was a significant main effect of group [*F*_(1, 42)_ = 6.82, *p* = 0.012] and a significant interaction effect of group × time for all matches [*F*_(2, 73)_ = 3.92, *p* = 0.024]. The dyads of the intervention group spent more time than the control group in states of match after both 3 months of intervention [*b* = 0.11, *t*_(67)_ = 2.11, *p* = 0.039] and 6 months [*b* = 0.16, *t*_(77)_ = 2.56, *p* = 0.012]. Furthermore, the effect was greater from 3 to 6 months than from 6 to 9 months [*b* = 0.04, *t*_(77)_ = 0.77, *ns;* see Figure 3A].

There was also a significant main effect of group [*F*_(1, 42)_ = 6.97, *p* = 0.011] and a significant interaction effect of group × time for all mismatches [*F*_(2, 73)_ = 3.70, *p* = 0.029]. The dyads of the intervention group spent less time in states of mismatch from 3 to 9 months after both 3 months of intervention [*b* = −0.11, *t*_(67)_ = −2.04, *p* = 0.045] and 6 months of intervention than the control group[*b* = −0.16, *t*_(78)_ = −2.49, *p* = 0.015]. Furthermore, the effect was greater from 3 to 6 months than from 6 to 9 months [*b* = 0.04, *t*_(78)_ = 0.75, *ns*; see Figure 3B].

Finally, for what concerns capacity for repair, i.e., moving from states of mismatch to states of match, there were significant main effects of group [*F*_(1, 44)_ = 14.16, *p* = 0.000] and time [*F*_(1, 77)_ = 3.82, *p* = 0.026] and a significant interaction effect of group × time [*F*_(2, 77)_ = 8.19, *p* = 0.001]. The dyads of the intervention group compared to the dyads of the control group showed from 3 to 9 months an increase in the frequency of repair after both 3 months of intervention [*b* = 1.74, *t*_(70)_ = 3.08, *p* = 0.003] and 6 months [*b* = 2.44, *t*_(82)_ = 3.68, *p* = 0.000; see Figure 3C]. Furthermore, the effect was greater from 3 to 6 months than from 6 to 9 months [*b* = 0.70, *t*_(72)_ = 1.05, *ns*].

The profile of the control group was the converse of that of the intervention group since it showed an increase in the amount of time spent in states of mismatch and a decrease both in the amount of time spent in states of match and in the frequency of repair from mismatch to match from 3 to 9 months.

### The moderating effect of maternal attachment representations and reflective functioning on the effectiveness of intervention

Given that the scores of maternal reflective functioning were strongly associated to the type of maternal attachment (Fisher's exact test = 30.98; *p* = 0.000), in that the adolescent mothers with secure attachment had higher scores of reflective functioning than did the adolescent mothers with insecure attachment [*t*_(43)_ = 6.85; *p* = 0.000], it was decided to use only one of the two variables, the attachment model, as a possible moderator of the effectiveness of intervention.

To investigate whether the factor of maternal attachment representation was moderating the effect of the intervention, GLMMs were conducted that used AAI status (secure vs. insecure), group (intervention vs. control) and time as fixed effects. In particular, to evaluate the moderating effect of maternal attachment representations, the effect of interaction between group, time and quality of attachment were considered. Complete analysis was only possible for participants who did the AAI (*n* = 45).

Analysis indicated that there were no significant interaction effects between the effect of intervention and the quality of the maternal attachment model either for mother or infant styles measured with the Care-Index, for play with objects, affective coordination and repair evaluated with ICEP (see Table 4).

**Table 4:** **Main and interaction effects of the attachment model and effectiveness of intervention**.

		**Maternal attachment**			**Maternal attachment × Time × Group**		
		***F***	***df***	***p***	***F***	***df***	***p***
Care-index	Sensitivity	1.03	40	0.31	0.52	66	0.59
	Controlling	0.19	41	0.65	0.02	66	0.97
	Unresponsiveness	1.59	41	0.21	0.29	68	0.74
	Cooperative	0.42	40	0.51	1.24	67	0.29
	Compliant-compulsive	1.04	41	0.35	0.64	66	0.52
	Difficult	0.44	41	0.50	1.91	65	0.15
	Passive	0.53	40	0.46	1.27	66	0.28
ICEP	Infant orientation to objects offered by the mother	0.03	42	0.84	0.40	74	0.67
	Infant orientation to objects not offered by the mother	0.49	40	0.48	0.56	69	0.57
	Mother involvment in play	0.59	40	0.44	0.94	69	0.39
	Mother offer of object	0.03	39	0.84	0.25	69	0.77
	Infant positive-mother positive	0.74	39	0.39	0.38	70	0.68
	Infant negative-mother negative	0.66	44	0.41	0.63	75	0.53
	Infant neutral-mother neutral	1.16	37	0.29	0.15	68	0.85
	Infant positive-mother negative	1.60	46	0.21	1.01	76	0.36
	Infant positive-mother neutral	0.00	41	0.94	0.39	68	0.67
	Infant negative-mother positive	0.40	39	0.53	0.08	72	0.92
	Infant negative-mother neutral	0.14	39	0.70	1.21	72	0.30
	Infant neutral-mother positive	0.32	40	0.57	0.29	70	0.74
	Infant neutral-mother negative	1.47	34	0.23	1.94	62	0.15
	Match	3.73	35	0.06	0.12	65	0.88
	Mismatch	3.87	36	0.05	0.10	66	0.90
	Repair	0.40	39	0.52	0.02	69	0.97

*Degrees of freedom (df) and level of significance (p)*.

### A case history

To illustrate our way of working, we shall here describe in brief the intervention with a mother-infant couple. Sofia was 20 when she arrived at our service with her 2-month old infant, Marco. Sofia's condition was characterized by multiple risk factors due to her family history. Her parents divorced when she was young and in the following period her mother was imprisoned and her father abused her physicaly and psychologically. She then went to live with her maternal grandparents and four younger siblings. From that moment on Sofia had to take the place of her mother in looking after her siblings, often suffering anxiety when caring for them. After the birth of Marco, Sofia went to live with her partner and decided to stop looking after her siblings. This decision, however, gave rise to a strong sense of guilt which she struggled with.

Of particular use in understanding how Sofia had processed these events was the AAI conducted with her at infant 3 months. Sofia's attachment model was preoccupied with aspects of anger both toward her parents (E2; Main et al., 2002), even though Sophia displayed some awareness of her adverse experiences. In this context Marco's birth had particular significance for Sofia: she could at last look after her own needs and not those of her siblings. However, this desire clashed immediately with Marco's needs and the anxiety which Sofia experienced with her siblings emerged anew.

In the first video session the interaction between Sofia and Marco immediately seemed problematic. A style of interaction prevailed which oscillated between intrusive and non-responsive, while Marco expressed distress by crying and avoiding contact with his mother. Sofia also expressed difficulty in interacting with Marco by playing and sharing positive emotions. In the video interventions the team worked with Sofia both on the meaning of Marco's crying and on his attempts at communication. In the second 6 months, Sofia seemed to find it difficult to support Marco's desire to explore and to share activities with her. Thanks to the video sessions triadic interaction improved significantly.

The assessment conducted with the Care-Index and the ICEP before the intervention (2 months) and at the end (9 months) showed, for Sofia, a significant increase in sensitivity and a decrease in unresponsiveness and controlling style and, for Marco, an increase in cooperative style and a decrease in passive and difficult style. There had also been a fall in negative matches and an increase in positive matches and in the capacity for repair.

The counseling conducted with Sofia in parallel with the video intervention allowed her to address the sense of solitude that she had experienced with respect to being neglected by her mother and the anger she felt toward her. At the same time Sofia managed to make sense of the acute anxiety she had felt looking after her siblings and which she now felt from time to time caring for Marco.

At the end of the intervention Sofia began to make plans for herself and to consolidate the relationship with her partner who constantly supported her during the intervention. At 16 months Marco's attachment to his mother, assessed with the Strange Situation Procedure (Ainsworth et al., 1978), was secure, unlike that of Sofia which was insecure preoccupied at the beginning of the intervention. The chain of intergenerational transmission of attachment had therefore been broken. Likewise the absence of neglect and abuse in Sofia's caring for Marco bear witness to the fact that there had been no transmission to Marco of the adverse experiences suffered by Sofia.

The intervention thus achieved both aims of our program: to support the mother-infant relationship and to protect the growth of the adolescent mother. In this regard the relationship of trust which formed between Sofia and the two therapists (a psychologist and a psychomotrist) who followed her was very important, serving as secure base for her budding relationship with Marco.

## Discussion

The pilot study confirms the initial hypotheses, showing that after 3 and 6 months of intervention the PRERAYMI program, based on three strategies, video intervention, psychological counseling and developmental guidance, is effective both considering at a global level, the sensitivity of the mother and the cooperation of the infant, and considering at micro-analytic level, the affective coordination of the adolescent mother-infant dyads.

Analysis of styles of interaction between mother and infant assessed at a global level with the Care-Index scales shows that in the group of dyads with intervention the sensitivity of the mothers increases significantly both after 3 and 6 months of intervention, while there is a decrease in their Controlling style. On the contrary, in the control group there is a decrease in maternal Sensitivity style already after 3 months, while Controlling style remains high. Likewise, the Cooperative style of the infants of the intervention group increases significantly after 3 and 6 months and their Passive style decreases after 6 months, unlike the infants of the control group in which the Cooperative style remains stable and the Passive style increases after 6 months.

Furthermore, the intervention group at 9 months reaches an average score of 8.6 of sensitivity for the mothers and 8.2 of cooperativity for the infants, which indicates, according to Crittenden (1998), an adequate quality of mother and infant interaction, moving from the band of risk observed at the start of intervention to the band of adequacy at the end of intervention. In the control group, however, there was a decrease in sensitivity toward an average score of 4.0 for mothers and a decrease in cooperation toward an average score of 3.5 for infants. The control group dyads remain, therefore, in the same area of risk with respect to quality of interaction, defined by Crittenden as a band in “need of further intervention” (Crittenden, 1998), as that observed at infant 3 months.

This data indicates that intervention may be considered effective, given that it has been demonstrated that maternal sensitivity and low hostility are correlated with a positive outcome for the development of the infant at the level of both the quality of his attachment and psychopathological risk, while an association between low maternal sensitivity and control or hostility and psychopathological risk has been shown (Cumberland-Li et al., 2003; Mäntymaa et al., 2004; Lorber and Egeland, 2009; Haltigan et al., 2013). Furthermore, the increase in infant cooperation, understood as the capacity to interact with the mother and the decrease in passivity, understood as poor involvement and withdrawal from interaction, may be considered indicators of effectiveness. A number of studies have shown a correlation between maternal sensitivity and infant cooperation (Crittenden, 2008) and a positive association between infant cooperation and low psychopathological risk (Riva Crugnola et al., 2013a).

An increase in dyadic affective coordination was also observed in the intervention group. In the dyads with intervention after 3 and 6 months affective coordination increased both at the level of amount of time spent in states of affective coordination and amount of time spent in positive match. The amount of time spent in states of mismatch decreased. Lastly, there was an increase in capacity for repair. In the control group we see the contrary happening, with an increase in amount of time spent in states of mismatch and a decrease in amount of time spent in states of match; at the same time the frequency of repair decreases. The increase in affective coordination in the intervention dyads must be considered a key piece of data with respect to the effectiveness of intervention in that it is held by various researchers to be a particularly significant indicator with regard to the adequacy of the mother/infant relationship. See, in particular, the model of Tronick (2007) on mutual parent/infant regulation, which considers affective coordination and the capacity to repair errors in communication to be a central aspect of the functioning of a parent/infant dyad. Various studies show, in this regard, that in conditions of risk for parenthood (Riva Crugnola et al., 2013a), including maternal perinatal depression (Reck et al., 2011), there is less of this coordination than is the case with dyads without conditions of risk.

For what concerns individual mismatches, in the intervention dyads we see a decrease in the mismatch “Infant positive/Mother negative,” a mismatch which increases in the control group. The decrease is an indicator of effectiveness, given that this type of mismatch, involving negative states in the mother in the co-presence of positive states in the infant, is considered to be an expression of dysfunctional mother/infant communication (Lyons-Ruth, 2006).

The intervention was also effective for what concerns triadic involvement in play with objects by the mother/infant dyads who used the intervention. In the mothers after 3 and 6 months and in the infants after 6 months there was a greater increase in amount of time spent in mutual involvement in play than in the control group. This may also be considered an indicator of effectiveness since it indicates that at 9 months the infants of the intervention group showed that they had achieved to a greater extent than the control group the mother/infant/object triadic coordination typical of secondary intersubjectivity, demonstrated by the increase in the behavior “Orientation to Objects Offered by Mother.” This achievement may be connected to the increased capacity of the mothers of the intervention group to carry out a scaffolding function, demonstrated by the greater increase in these mothers in the behavior “Involvement in Play” of the infant, an increase which is lower in the mothers of the control group.

To sum up, the program has proven to be effective both at the level of mother/infant styles of interaction and at the level of mother/infant affective and play coordination. We may hypothesize that the use of different techniques—video intervention, counseling and developmental guidance—aimed at increasing the sensitivity of the mother, her capacity to regulate the infant's emotions and her capacity to reflect on her own mental states and on those of the infant may explain this efficacy. Indeed, it is known that maternal sensitivity and reflective capacity are correlated (Slade et al., 2005a).

Finally, for what concerns comparison of the results after 3 and 6 months of intervention it is interesting to note that the majority of the variables change for the better already after 3 months of intervention and this increase is greater in that period than it is in the subsequent 3 months. See in particular, in this regard, at an individual level, maternal sensitivity and control and infant cooperation and at dyadic level, the amount of time spent in states of affective coordination and in states of positive match, the amount of time spent in mismatch Infant positive/Mother negative and the capacity for repair. These data indicate the importance of beginning intervention with adolescent mothers in the first months after birth. It may be hypothesized in this regard that, by acting early and intensively with different strategies, the intervention has an effect right from the first months, helping to change inadequate parental behaviors, increasing infant cooperation and improving dyadic affective coordination. These changes remain stable also after 3 months right to the end of intervention. Recent meta-analyses (Geeraert et al., 2004; Nievar et al., 2010) have demonstrated that intervention programs which provide for very frequent meetings in the first months of intervention are twice as effective as programs with less frequent meetings.

Our results are also in line with studies that have showed that attachment-based programs for adolescent mothers which use video-feedback are effective in improving the quality of maternal parenting (Moran et al., 2005; Slade et al., 2005b; McDonald et al., 2009), as well as with studies on the use of video-feedback in attachment-based intervention for at risk parents (Steele et al., 2010).

It is important to stress, however, that our study is more exhaustive than previous studies. It assesses the effectiveness of intervention not only with respect to maternal sensitivity but to the cooperative contribution of the infant too. Furthermore, it also considers dyadic affective coordination as an outcome variable. Our study, therefore, demonstrates the effectiveness of intervention since it helps increase not only maternal sensitivity but also infant cooperation, at the same time influencing both partners' capacity for mutual emotion regulation. Only one attachment-based study for adolescent mothers (Mayers et al., 2008) has assessed infant as well as maternal contribution and no study has considered dyadic emotion regulation.

Another important result is that there were no significant differences with respect to the effectiveness of intervention in relation to maternal attachment models. In other conditions of risk for the parent/infant relationship, such as that of insecure maternal representations, the program therefore maintained its effectiveness. In fact it is well-known that insecure maternal attachment models are associated with a lower level of adequacy in the mother/infant relationship, at the level of emotional attunement (Haft and Slade, 1989; De Oliveira et al., 2005), emotional availability (Biringen et al., 2000) and dyadic emotion regulation (Riva Crugnola et al., 2013a). In other attachment-based programs maternal disorganization with respect to attachment (Moran et al., 2005) was shown to be a factor which made intervention ineffective. Our data, however, are in line with a recent study which showed that an attachment-based intervention program for high-risk dyads was effective with both secure and insecure mothers (Pillhofer et al., 2014).

Our study has a number of limits. One of these is that it is a pilot study which therefore examined the effectiveness of intervention only at its conclusion, without there yet being sufficient data on the two follow-up stages, relating to assessment of infant attachment at 14 months and assessment of their possible psychopathological risk at 24 months. The small number of participants also reduces the possibility of generalizing the results. Another limit is the non-randomized assignment of participants to the intervention and control groups. It must, however, be considered that at the beginning of the intervention the participants did not differ significantly either socio-demographically or in their styles of interaction and regulation measured with the Care-Index and ICEP.

Future objectives of the study, in addition to that of continuing with the follow up stages, will be (by increasing the number of participants) to study other variables which may affect outcome. Variables of particular interest could be a previous history of abuse, something which is very frequent in the adolescent mothers we follow, and symptoms of perinatal depression in the mothers for whom the intervention is intended. A further objective could be to assess any variations in the mothers' capacity of reflective functioning, measuring it pre and post-intervention.

## Author contributions

All authors listed, have made substantial, direct and intellectual contribution to the work, and approved it for publication.

### Conflict of interest statement

The authors declare that the research was conducted in the absence of any commercial or financial relationships that could be construed as a potential conflict of interest.
